# Variation in quality of preventive care for well adults in Indigenous community health centres in Australia

**DOI:** 10.1186/1472-6963-11-139

**Published:** 2011-06-01

**Authors:** Ross S Bailie, Damin Si, Christine M Connors, Ru Kwedza, Lynette O'Donoghue, Catherine Kennedy, Rhonda Cox, Helen Liddle, Jenny Hains, Michelle C Dowden, Hugh P Burke, Alex Brown, Tarun Weeramanthri, Sandra Thompson

**Affiliations:** 1 Menzies School of Health Research, Charles Darwin University, Darwin NT, Australia; 2 School of Medicine, University of Queensland, Brisbane QLD, Australia; 3 Northern Territory Department of Health and Families, Darwin NT, Australia; 4 Queensland Department of Health, Cairns QLD, Australia; 5 Maari Ma Health Aboriginal Corporation, Broken Hill NSW, Australia; 6 Curtin University, Perth WA, Australia; 7 Baker IDI Heart and Diabetes Institute (Alice Springs), Alice Springs NT, Australia; 8 Western Australia Department of Health, Perth WA, Australia; 9 Aboriginal Health Council of Western Australia, Perth WA, Australia

## Abstract

**Background:**

Early onset and high prevalence of chronic disease among Indigenous Australians call for action on prevention. However, there is deficiency of information on the extent to which preventive services are delivered in Indigenous communities. This study examined the variation in quality of preventive care for well adults attending Indigenous community health centres in Australia.

**Methods:**

During 2005-2009, clinical audits were conducted on a random sample (stratified by age and sex) of records of adults with no known chronic disease in 62 Indigenous community health centres in four Australian States/Territories (sample size 1839). Main outcome measures: i) adherence to delivery of guideline-scheduled services within the previous 24 months, including basic measurements, laboratory investigations, oral health checks, and brief intervention on lifestyle modification; and ii) follow-up of abnormal findings.

**Results:**

Overall delivery of guideline-scheduled preventive services varied widely between health centres (range 5-74%). Documentation of abnormal blood pressure reading ([greater than or equal to]140/90 mmHg), proteinuria and abnormal blood glucose ([greater than or equal to]5.5 mmol/L) was found to range between 0 and > 90% at the health centre level. A similarly wide range was found between health centres for documented follow up check/test or management plan for people documented to have an abnormal clinical finding. Health centre level characteristics explained 13-47% of variation in documented preventive care, and the remaining variation was explained by client level characteristics.

**Conclusions:**

There is substantial room to improve preventive care for well adults in Indigenous primary care settings. Understanding of health centre and client level factors affecting variation in the care should assist clinicians, managers and policy makers to develop strategies to improve quality of preventive care in Indigenous communities.

## Background

As part of the response to high levels of chronic disease among Indigenous Australians[1], there has been increasing emphasis in recent years on delivery of preventive services in Indigenous primary health care services. This includes development and distribution of evidence-based, Indigenous population specific preventive care guidelines [2], introduction of Medicare reimbursed biennial health checks for Indigenous adults aged 15 years or over [3], and the newly released National Preventative Health Strategy which specifies targets and actions for multifaceted preventive care to "close the gap" in life expectancy between Indigenous and other Australians [4].

Previous research provides limited information on delivery of preventive care in Indigenous primary care settings. Studies conducted in the Northern Territory (NT) have documented substantial deficiencies in delivery of preventive care to Indigenous adults in rural and remote communities: on average only 40-50% of preventive services were delivered in line with the best practice guidelines [5,6]. Studies in Indigenous communities in Queensland (QLD) [7,8] have not included data on the proportion of community members who received health checks. A national study using Medicare data showed that 3% of Indigenous Australians aged 55 years or over attending GPs had documented use of specific Medicare items for health checks [9]. However, the study appeared to underestimate the uptake of preventive health checks as many Indigenous primary care services do not use Medicare items when delivering services to clients. Thus there is substantial potential to improve the quality of information on the delivery of preventive services to Indigenous people for the purpose of informing implementation of the National Preventative Health Strategy.

The Audit and Best-practice for Chronic Disease Extension (ABCDE) project is a national quality improvement initiative which aims to improve quality of care in a range of priority aspects of Indigenous primary health care, including chronic disease care, preventive care, and maternal and child health care [10]. During the past five years over 60 Indigenous community health centres from four States/Territories (NT, Far West New South Wales (NSW), Western Australia (WA) and North QLD) have formally participated in this project. The ABCDE data provide a unique opportunity to improve understanding of delivery of preventive care in Indigenous primary health care settings, and, importantly, to develop and implement strategies for improvement. This paper reports baseline data on delivery of preventive care in Indigenous community health centres participating in ABCDE with a focus on variation in quality of care between services and across different participating regions, and identifies the various factors associated with these variations, both at the health centre level and at the individual level.

## Methods

**Figure 1:**
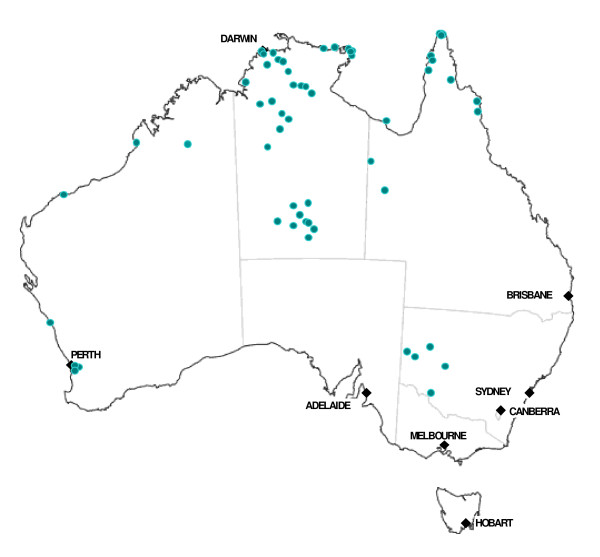
**Geographic distribution of participating Aboriginal community health centres in the study**.

Participation by health centres was from five regions where we had established project hub coordinators (Figure 1 and Table 1). On a voluntary basis, health centre managers or staff made a request for their centre to join the project after receiving information through invitation letters, word of mouth or meeting presentations. Sixty six (66) health centres formally participated in the ABCDE project. Four (4) of these health centres did not have at least part time access to a GP and were excluded from the analysis for this paper.

**Table 1:** Characteristics of participating health centres and adults

Characteristic	NT Top End	NT Central Australia	Far West NSW	WA	North QLD	Total
**Community health centres**	**24**	**9**	**5**	**7**	**17**	**62**
Locations						
City	1 (4%)	1 (11%)	0 (0%)	3 (43%)	0 (0%)	5 (8%)
Regional town	6 (25%)	0 (0%)	4 (80%)	2 (28%)	0 (0%)	12 (19%)
Remote community	17 (71%)	8 (89%)	1 (20%)	2 (29%)	17 (100%)	45 (73%)
Health service governance						
Government funded/operated	10 (42%)	5 (56%)	0 (0%)	1 (14%)	17 (100%)	33 (53%)
Managed by local or regional Indigenous committee or board	14 (58%)	4 (44%)	5 (100%)	6 (86%)	0 (0%)	29 (47%)
General practice accreditation status						
Currently accredited	9 (38%)	3 (33%)	1 (20%)	2 (29%)	5 (29%)	19 (31%)
Not accredited	15 (62%)	6 (67%)	4 (80%)	5 (71%)	12(71%)	43 (69%)
Sizes of populations served						
≤ 500	9 (37%)	4 (44%)	1 (20%)	0 (0%)	6 (35%)	20 (32%)
501-999	6 (25%)	4 (45%)	3 (60%)	0 (0%)	4 (24%)	17 (28%)
≥ 1000	9 (38%)	1 (11%)	1 (20%)	7 (100%)	7 (41%)	25 (40%)
**Participating adults**	**708**	**267**	**150**	**210**	**504**	**1839**
Mean age (years)	32	31	33	32	33	32
Males	49%	49%	50%	50%	50%	49%
Indigenous status						
Indigenous	94%	95%	43%	87%	91%	89%*
Non-Indigenous	4%	1%	50%	10%	1%	7%*
Not stated	2%	4%	7%	3%	8%	4%
Attended health centre within the past 24 months (range between health centres)	93% (50%-100%)	97% (90-100%)	75% (30%-93%)	99% (93%-100%)	90% (53%-100%)	92%* (30%-100%)
Reasons for last attendance						
Well person's check	13%	10%	4%	3%	4%	8%*
Acute care	58%	56%	42%	59%	53%	55%*
Immunisation	2%	2%	4%	1%	12%	5%
Sexual health	7%	9%	4%	3%	6%	6%
Mental health	1%	1%	5%	0%	2%	1%
others	19%	22%	41%	34%	23%	25%*
Key responsible health providers during the last attendance						
Nurses	48%	64%	39%	13%	55%	47%*
Aboriginal health workers	26%	14%	7%	13%	15%	18%*
Doctors	14%	12%	40%	62%	9%	20%*
Specialists	1%	1%	4%	2%	2%	1%
Others	11%	9%	10%	10%	19%	14%
Documented as a smoker	30%	23%	27%	28%	29%	28%
Documented alcohol misuse	15%	15%	27%	12%	40%	23%*
Documented organic complications of alcohol misuse	5%	7%	3%	4%	13%	7%*

* P < 0.05 for comparison between regions.

Baseline audits of preventive care were completed during 2005-2009. Audits covered both paper-based and electronic clinical records. The records of health centre clients who met all of the following criteria were eligible for inclusion: 1) aged between 15 and 54 years; 2) resident in the community for at least 6 of the last 12 months; 3) not having a diagnosis of diabetes, hypertension, ischaemic heart disease, rheumatic heart disease, renal disease or other major chronic illness; and 4) not pregnant or post partum at the time of the audit. Eligibility was verified by checking an up-to-date population list of health centre clients with assistance from health centre staff who knew the community and health centre well. A sample of 30 records, stratified by sex and age groups (15-24; 25-39; and 40-54 years), was selected randomly from records of eligible clients in each health centre. Thus, each random sample comprised 5 males and 5 females in each of the age groups. In communities where there were fewer than 5 people in a sex- and age-specific group, all eligible people in that group were included.

The audit measured 16 selected service items (see Table 2) which the preventive health care guidelines recommend for delivery every year or every two years for all Indigenous well adults aged 15-54 years [2,11]. A summary of detailed guideline recommendations in relation to the 16 service items is presented in Table 3. We adopted a minimum approach to assess whether these services were delivered on a two-yearly basis. A service was assessed as delivered if there was a clear record of delivery of the service at least once within the previous 24 months. The overall adherence to delivery of scheduled services for each adult was calculated by dividing the sum of services delivered by 16 (for females) or 15 (for males - pap smear excluded), and expressing this as a percentage. For example, if there were 6 services assessed as delivered for a male client, the overall adherence to delivery of services for the client was 40% (6/15), interpreted as "40% of guidelines-scheduled preventive services were delivered to the client". Health centre-level adherence was computed as the mean of individual adherence to delivery at each centre. For each individual service item, a percentage (from 0 to 100%) was calculated at each health centre to represent "% of patients who received the specific preventive service".

**Table 2:** Mean adherence to delivery of scheduled services for 62 community health centres within previous 24 months

	NT Top End	NT Central Australia	Far West NSW	WA	North QLD	Total
	
Process item	Mean percentages ± SE (range between health centres)					
**Total No. of centres | adults**	24 | 708	9 | 267	5 | 150	7 | 210	17 | 504	62 | 1839
**Basic measurements**						
Weight	56% ± 5% (10%-100%)	58% ± 7% (18%-83%)	28% ± 7% (0%-40%)	52% ± 9% (17%-73%)	61% ± 5% (28%-87%)	55% ± 3%* (0%-100%)
Height	40% ± 6% (3%-100%)	40% ± 9% (4%-77%)	23% ± 6% (0%-40%)	47% ± 10% (3%-70%)	39% ± 3% (17%-63%)	39% ± 3% (0%-100%)
BMI	26% ± 6% (0%-87%)	25% ± 9% (0%-77%)	15% ± 6% (0%-33%)**	27% ± 8% (3%-60%)	15% ± 4% (0%-57%)	22% ± 3% (0%-87%)
Waist circumference	26% ± 5% (0%-85%)	28% ± 8% (0%-60%)	15% ± 6% (0%-33%)**	11% ± 7% (0%-57%)	17% ± 3% (0%-57%)	21% ± 3% (0%-85%)
Blood pressure	71% ± 4% (23%-100%)	75% ± 6% (33%-90%)	55% ± 9% (23%-73%)	65% ± 9% (27%-93%)	76% ± 4% (41%-97%)	71% ± 3% (23%-100%)
**Laboratory investigations**						
Urinalysis	43% ± 5% (8%-97%)	46% ± 8% (11%-77%)	13% ± 5% (7%-30%)	34% ± 10% (13%-80%)	42% ± 4% (21%-80%)	40% ± 3%* (7%-97%)
Blood glucose level	56% ± 5% (13%-97%)	56% ± 9% (17%-83%)	35% ± 8% (7%-53%)**	53% ± 8% (20%-77%)	65% ± 4% (41%-90%)	56% ± 3% (7%-97%)
Pap smear (women only)	42% ± 5% (0%-80%)	44% ± 8% (6%-93%)	28% ± 9% (0%-53%)	28% ± 7% (0%-56%)	42% ± 4% (13%-60%)	40% ± 3% (0%-93%)
STI: PCR for gonorrhoea & chlamydia	44% ± 6% (0%-93%)	57% ± 7% (20%-83%)	5% ± 2% (0%-10%)	25% ± 10% (0%-73%)	43% ± 5% (4%-87%)	40% ± 3% * (0%-93%)
STI: Syphilis serology	36% ± 5% (0%-93%)	57% ± 8% (18%-90%)	4% ± 3% (0%-13%)	16% ± 9% (0%-57%)	35% ± 5% (0%-90%)	34% ± 3%* (0%-93%)
**Oral health check**	18% ± 3% (0%-63%)	16% ± 7% (0%-67%)	11% ± 5% (0%-23%)	17% ± 9% (0%-67%)	10% ± 4% (0%-50%)	15% ± 2% (0%-67%)
**Brief intervention/advice**						
Smoking	21% ± 4% (0%-73%)	35% ± 8% (3%-70%)	20% ± 6% (10%-43%)	17% ± 6% (3%-53%)	39% ± 4% (10%-75%)	28% ± 3%* (0%-75%)
Nutrition	17% ± 5% (0%-81%)	36% ± 8% (7%-67%)	13% ± 4% (3%-23%)	12% ± 6% (0%-47%)	34% ± 4% (8%-63%)	24% ± 3%* (0%-81%)
Alcohol	20% ± 4% (0%-73%)	37% ± 7% (13%-67%)	18% ± 6% (7%-40%)	17% ± 6% (3%-53%)	41% ± 4% (17%-75%)	28% ± 3%* (0%-75%)
Physical activity	17% ± 5% (0%-88%)	36% ± 8% (0%-67%)	11% ± 4% (3%-27%)	14% ± 7% (3%-53%)	31% ± 4% (0%-63%)	23% ± 3%* (0%-88%)
Mood (emotional well-being)	17% ± 5% (0%-81%)	28% ± 7% (0%-67%)	15% ± 8% (3%-43%)**	15% ± 6% (3%-47%)	18% ± 4% (0%-50%)	18% ± 2% (0%-81%)
**Overall adherence**	34% ± 4% (8%-74%)	42% ± 7% (15%-71%)	19% ± 5% (5%-32%)	28% ± 6% (13%-59%)	38% ± 3% (21%-65%)	34% ± 3% (5%-74%)

* P < 0.05 for comparison between regions.

** indicating statistically significant differences (P < 0.05) between Indigenous and non-Indigenous adults in Far West NSW, as detailed below: BMI (Indigenous 23% vs non-Indigenous 8%, P = 0.03); waist (22% vs 8%, P = 0.04); blood glucose level (48% vs 25%, P = 0.04); and mood (22% vs 11%, P = 0.02).

Percentages are based on standard stratified samples from each health centre with equal numbers of males and females in three age bands and are not weighted for population size.

**Table 3:** Recommended schedule for the adult well person's health check for Indigenous people

Service item	Guideline	Frequency	Starting age
**Basic measurements**			
Weight	A	Yearly	10 years
	B	Yearly	15 years
Height	B	1^st ^visit	
BMI	A	Yearly	10 years
	B	Yearly	15 years
Waist circumference	A	Yearly (6-monthly in obesity/overweight)	18 years
	B	Yearly	15 years
Blood pressure	A	Check every visit, at least yearly	18 years or earlier
	B	Yearly	15 years
**Laboratory investigations**			
Urinalysis	A	Yearly	15-18 years
	B	Yearly	15 years
Blood glucose level	A	1-2 yearly	15-18 years in high prevalence
	B	Yearly	15 years
Pap smear (women only)	A	2-yearly	Women aged 18-70
STI: PCR for gonorrhoea & chlamydia	A	1-2 yearly	< 25 years for sexually active men and women
	B	Yearly	15 years
STI: Syphilis serology	A	1-2 yearly	15-30 years
	B	Yearly	15 years
**Oral health check**	A	Yearly	All ages
**Brief intervention/advice**			
Smoking	A	Yearly	10 years
	B	Yearly	15 years
Nutrition	B	Yearly	15 years
Alcohol	A	Yearly	14-15 years
	B	Yearly	15 years
Physical activity	B	Yearly	15 years
Mood (emotional well-being)	A	Every visit	15 years

A - National guide to a preventive health assessment in Aboriginal and Torres Strait Islander peoples [2].

B - CARPA standard treatment manual: a clinic manual for primary health care practitioners in remote and rural communities in Central and Northern Australia [11].

Clinical records were also audited for evidence of abnormal blood pressure readings, positive protein in urine and abnormal blood glucose readings. For any abnormality found we checked for a record of a follow-up as outlined in Table 4. A percentage was calculated for each health centre to represent "% of adults who had appropriate follow-up of abnormal findings".

**Table 4:** Follow-up of abnormal clinical findings at participating health centres

	NT Top End	NT Central Australia	Far West NSW**	WA		North QLD		Total
	
Abnormal findings and follow ups	Mean percentages ± SE (range between health centres)							
**Total No. of centres | adults**	24 | 708	9 | 267	5 | 150		7 | 210		17 | 504	62 | 1839
Total number of adults with recorded BP readings	507	201	83		137		379	1307
Percentage of adults with BP readings ≥ 140/90 mmHg	9% ± 2% (0%-28%)	8% ± 2% (4%-24%)	24% ± 7% (0%-43%)		9% ± 2% (0%-16%)		27% ± 6% (7%-100%)	15% ± 2%* (0%-100%)
Percentage of adults with an abnormal BP who had a repeated BP check and/or subsequent management plan	9% ± 5% (0%-100%)	13% ± 11% (0%-100%)	29% ± 17% (0%-67%)		21% ± 16% (0%-100%)		32% ± 6% (0%-100%)	19% ± 4%* (0%-100%)
Total number of adults with recorded urine dipstick tests	303	123	19		72		212	729
Percentage of adults having positive protein in urine tests	16% ± 3% (0%-50%)	17% ± 6% (0%-60%)	17% ± 9% (0%-50%)		17% ± 7% (0%-50%)		30% ± 6% (0%-92%)	20% ± 2%* (0%-92%)
Percentage of adults with an abnormal urine test who had a subsequent ACR test and/or management plan	20% ± 8% (0%-100%)	38% ± 18% (0%-100%)	0% (-)		0% (-)		37% ± 8% (0%-100%)	25% ± 5% (0%-100%)
Total number of adults with recorded blood glucose readings	396	150	53		111		319	1029
Percentage of adults with glucose readings ≥ 5.5 mmol/L	33% ± 5% (0%-89%)	35% ± 8% (10%-67%)	23% ± 12% (0%-67%)		19% ± 5% (0%-35%)		53% ± 5% (10%-94%)	37% ± 3%* (0%-94%)
Percentage of adults with an abnormal glucose tests who had a repeated glucose test and/or subsequent management plan	22% ± 6% (0%-88%)	11% ± 8% (0%-75%)	19% ± 10% (0%-33%)		14% ± 9% (0%-50%)		23% ± 6% (0%-100%)	19% ± 3% (0%-100%)

* P < 0.05 for comparison between regions.

** Further analyses showed no statistically significant differences in measures between Indigenous and non-Indigenous adults in Far West NSW.

Percentages are based on standard stratified samples from each health centre with equal numbers of males and females in three age bands and are not weighted for population size.

### Statistical analysis

The quality of preventive care was measured in terms of adherence to delivery of scheduled services and follow-up of abnormal findings. Treating health centres as the unit of analysis, we compared the quality of care (based on mean percentages) between regions using linear regression models (Tables 2 and 4). Centre percentages and mean percentages are unweighted.

When treating individual clients as the unit of analysis, our data had inherent multilevel, dependency structure, as preventive care data collected at the individual client level (level 1) were clustered within health centres (level 2). Two-level random effects regression models (linear or logistic) [12] were used to 1) quantify the amount of variation attributable to health centre and individual level characteristics (Table 5); and 2) examine associations of specific factors with quality of preventive care (Tables 6 and 7), as outlined below:

**Table 5:** Percent of total variation attributable to health centre level and individual patient level characteristics

	Percent of total variation in outcome measures attributable to:	
	
Outcome measure	Health centre level characteristics	Individual client level characteristics
**Adherence to delivery of preventive services**		
Basic measurements done		
Weight	29%	71%
Height	30%	70%
BMI	47%	53%
Waist circumference	42%	58%
Blood pressure	22%	78%
Laboratory investigations done		
Urinalysis	24%	76%
Blood glucose level	21%	79%
Pap smear (women only)	20%	80%
STI: PCR for gonorrhoea & chlamydia	39%	61%
STI: Syphilis serology	43%	57%
Oral health check done	44%	56%
Brief interventions/advice done		
Smoking	28%	72%
Nutrition	35%	65%
Alcohol	30%	70%
Physical activity	42%	58%
Mood (emotional well-being)	34%	66%
Overall adherence	31%	69%
**Follow up of abnormal findings**		
Proportion of adults with abnormal BP who had repeated BP check or documented plan to repeat BP	13%	87%
Proportion of adults with abnormal urine tests who had ACR tests or documented plan to test ACR	33%	67%
Proportion of adults with abnormal glucose tests who had repeated glucose tests or documented plan to repeat	38%	62%

**Table 6:** Associations of health centre level and individual level factors with adherence to delivery of scheduled preventive services

		Dependent variable	
		
Characteristic (independent variables)	Adherence to delivery (%)	Unadjusted Coefficients (95% CI)	Adjusted Coefficients* (95% CI)
**Regions**			
NT Top End	34%	Referent	Referent
NT Central Australia	42%	8% (-6%,23%)	7% (-5%,19%)
Far West NSW	19%	**-15% (-26%,-4%)**	**-14% (-27%,-2%)**
WA	28%	-6% (-19%,7%)	3% (-12%,18%)
North QLD	37%	3% (-7%,13%)	9% (-1%, 18%)
**Health centre level characteristics**			
Locations			
City (n = 150)	23%	Referent	Referent
Regional town (n = 360)	25%	2% (-7%,12%)	9% (-6%,24%)
Remote community (n = 1329)	38%	**15% (7%,22%)**	**18% (3%,33%)**
Health service governance			
Government (n = 980)	33%	Referent	Referent
Indigenous committee or board (n = 859)	35%	2% (-7%, 11%)	**13% (3%,24%)**
General practice accreditation status			
Not accredited (n = 1263)	33%	Referent	Referent
Currently accredited (n = 576)	36%	3% (-5%,12%)	6% (-1%,13%)
Sizes of populations served			
≤ 500 (n = 562)	42%	Referent	Referent
501-999 (n = 507)	34%	-8% (-20%,4%)	-5% (-15%,5%)
≥ 1000 (n = 770)	28%	**-14% (-24%,-4%)**	**-10% (-19%,-1%)**
**Individual level characteristics**			
Age (years)			
15-24 (n = 627)	32%	Referent	Referent
25-39 (n = 660)	37%	**5% (2%,9%)**	**6% (2%,9%)**
40-54 (n = 552)	33%	1% (-2%,4%)	3% (-1%,6%)
Sex			
Males (n = 909)	31%	Referent	Referent
Females (n = 930)	37%	**6% (3%,9%)**	**6% (3%,8%)**

R^2 ^of the model**		13%	

* Calculated using two-level random effects linear regression models, with adjustment of other variables in the table.

Coefficients significant at 0.05 level are shown in bold.

** R^2 ^represents the proportion of the total variability in the dependent variables that were explained by all independent variables listed in the table.

**Table 7:** Associations of health centre level and individual level factors with follow-up of abnormal findings

	Dependent variables								
	
Characteristic (independent variables)	Follow-up of abnormal BP (%)	Unadjusted Risk Ratios (95% CI)	Adjusted Risk Ratios* (95% CI)	Follow-up of abnormal urine tests (%)	Unadjusted Risk Ratios (95% CI)	Adjusted Risk Ratios* (95% CI)	Follow-up of abnormal glucose tests (%)	Unadjusted Risk Ratios (95% CI)	Adjusted Risk Ratios* (95% CI)
**Regions**									
NT Top End	13%	Referent	Referent	28%	Referent	Referent	29%	Referent	Referent
NT Central Australia	13%	0.9 (0.2,3.2)	1.5 (0.3,4.6)	40%	1.4 (0.5,2.7)	2.3 (0.9,3.2)	18%	0.6 (0.1,2.3)	0.6 (0.1,2.2)
Far West NSW	28%	2.1 (0.4,5.4)	0.9 (0.2,3.6)	0%	-	-	13%	0.5 (0.1,1.7)	0.5 (0.1,1.5)
WA	15%	1.2 (0.2,3.9)	1.2 (0.1,5.1)	0%	-	-	13%	0.5 (0.1,1.6)	0.6 (0.2,1.5)
North QLD	26%	2.0 (0.7,4.3)	**4.1 (1.6,6.3)**	42%	1.5 (0.8,2.3)	1.9 (0.8,2.9)	18%	0.6 (0.2,1.5)	0.8 (0.2,1.9)
**Health centre level characteristics**									
Locations									
City	0%	-	-	17%	Referent	Referent	20%	Referent	Referent
Regional town	25%	Referent	Referent	39%	2.3 (0.4,5.1)	4.7 (0.5,5.8)	15%	0.8 (0.2,1.9)	0.7 (0.2,1.9)
Remote community	22%	0.9 (0.4,1.7)	0.6 (0.1,2.2)	33%	2.0 (0.3,4.7)	2.3 (0.1, 5.6)	22%	1.1 (0.4,2.3)	0.9 (0.2,2.4)
Health service governance									
Government funded/operated	22%	Referent	Referent	38%	Referent	Referent	18%	Referent	Referent
Managed by local or regional Indigenous committee or board	21%	0.9 (0.5,1.8)	2.5 (0.8,4.0)	22%	0.6 (0.2,1.2)	1.1 (0.4,1.9)	27%	1.5 (0.5,3.2)	1.5 (0.4,3.8)
General practice accreditation status									
Not accredited	25%	Referent	Referent	37%	Referent	Referent	23%	Referent	Referent
Currently accredited	15%	0.6 (0.3,1.1)	0.5 (0.2,1.1)	25%	0.7 (0.3,1.3)	0.4 (0.1,1.1)	16%	0.7 (0.3,1.4)	0.8 (0.3,1.5)
Sizes of populations served									
≤ 500	31%	Referent	Referent	28%	Referent	Referent	30%	Referent	Referent
501-999	19%	0.6 (0.3,1.3)	0.5 (0.2,1.1)	34%	1.2 (0.6,2.1)	1.0 (0.5,1.8)	19%	0.7 (0.2,1.7)	0.7 (0.3,1.6)
≥ 1000	18%	0.6 (0.3,1.1)	0.8 (0.3,1.5)	36%	1.3 (0.6,2.2)	2.2 (0.9,3.0)	15%	0.5 (0.2,1.3)	0.6 (0.2,1.3)
**Individual level characteristics**									
Age (years)									
15-24	17%	Referent	Referent	36%	Referent	Referent	15%	Referent	Referent
25-39	19%	1.1 (0.4,2.5)	1.2 (0.4,2.7)	31%	0.9 (0.5,1.4)	0.9 (0.5,1.4)	25%	1.7 (0.9,2.7)	1.8 (0.9,2.9)
40-54	25%	1.5 (0.6,2.8)	1.6 (0.7,3.1)	32%	0.9 (0.4,1.5)	0.9 (0.3,1.7)	22%	1.5 (0.9,2.3)	1.7 (0.9,2.8)
Sex									
Males	22%	Referent	Referent	34%	Referent	Referent	20%	Referent	Referent
Females	21%	1.0 (0.6,1.5)	0.9 (0.5,1.4)	33%	1.0 (0.6,1.5)	0.9 (0.5,1.5)	22%	1.1 (0.7,1.6)	1.1 (0.8,1.6)

* Calculated using two-level random effects logistic regression models, with adjustment of other variables in the table. Odd Ratios generated from the models were converted into risk ratios using a published formula.[14]

Risk ratios significant at 0.05 level are shown in bold.

1) Adherence to delivery of services and follow up of abnormal findings were treated as dependent variables in the random effects models respectively. We constructed a two-level (health centre and client levels) random intercept model with no explanatory variables (also known as an empty model) [13]. In the context of multilevel modelling, the empty model provides an estimate of the basic partition of the variability in the data between the two levels. Based on the model, an intra-class correlation coefficient (rho in Stata [12]) between two randomly drawn individuals in a given health centre was estimated. The intra-class correlation coefficient can also be interpreted as the fraction of total variability in the dependent variable that is due to health centre level characteristics. The remaining variation is attributable to client level characteristics. The term "characteristics" used here refers to measured and un-measured factors at the health centre and client levels.

2) Using two-level random effects regression models, we also tested associations of specific factors at health centre (location, health service governance, accreditation status and population size) and individual levels (age and sex) with the quality of preventive care.

We obtained approval from formally constituted Human Research Ethics Committees (HREC, including Indigenous health research committees where such arrangements were in place) in each region in which the project operated, including the NT Department of Health & Community Services and Menzies School of Health Research HREC, the Central Australian HREC, the Western Australian Aboriginal Health Information and Ethics Committee, the Macquarie and Far West Area Health Services HREC, and the Townsville Health Service District HREC.

## Results

Of 62 participating health centres, 47% were managed by a local or regional Aboriginal committee (board), with the remainder government funded/operated (Table 1). Sixty nine percent of centres did not have formal general practice accreditation and most (60%) served populations of less than 1000 people.

Records of 1839 well adults were audited (Table 1). The mean age of these adults was 32 years and 49% were men. Around 90% or more of records from the NT, WA and North QLD centres were for Indigenous people compared to 43% from Far West NSW centres. Twenty eight percent of adults were documented as smokers and 23% had documented alcohol misuse. Ninety two percent of participants had a record of health centre attendance within the previous 24 months, with acute care the main reason for attendance and nurses as predominant health providers.

Overall delivery of scheduled services was 34% (Table 2), with substantial variation in this measure between health centres (range 5-74%) and moderate variation between regions (range 19-42%).

For specific preventive services, variation in delivery was evident across different categories of services, different regions, and different health centres (Table 2). Overall, adherence was relatively high for weight and blood pressure measurement and blood glucose testing (50-70%), followed by height measurement, urinalysis, pap smear and STI screening (30-40%), and waist circumference measurement and brief intervention/counselling on lifestyle modification (20-30%). Less attention was paid to oral health checks and brief intervention or counselling regarding emotional well being (15-18%). However, the range between health centres for delivery of almost all of these services was from 0- > 80%.

Analyses of preventive service delivery between Indigenous and non-Indigenous adults in Far West NSW health centres showed no statistical difference in overall service delivery between the two groups. However, Indigenous adults were more likely to receive services related to BMI and waist circumference measurements, blood glucose testing and emotional wellbeing counselling.

On average, health centre-level documentation of an abnormal blood pressure reading (≥ 140/90 mmHg) was found in 15% of adults, proteinuria in 20%, and abnormal blood glucose (≥ 5.5 mmol/L) in 37% (Table 4). However, the range between health centres for these measures were 0-100%, 0-92% and 0-94% respectively. North Queensland health centres had higher rates of abnormal blood pressure, proteinuria and abnormal blood glucose compared with other regions (P < 0.05 for comparison with NT Top End). Of those with identified abnormal clinical findings, overall about 20-30% had a documented follow up check/test or management plan, but the range between services was 0-100%.

Client level characteristics accounted for a large proportion of the variation in delivery of services and in follow up of abnormal findings: 69% for overall adherence to delivery of scheduled services (with a range of 53-79% for specific services); and between 62-87% for follow up of abnormal findings (Table 5).

Age and sex were both independently associated with overall delivery of services, with higher rates of delivery in the 25-39 year age group and in women (Table 6). Health centre level factors which were independently associated with higher level of delivery of services were location (remote community vs city), community population size (≤ 500 vs ≥ 1000), region (Top End vs FW NSW) and governance (Indigenous committee/board operated vs government).

For follow-up of abnormal findings, North Queensland had higher rates of follow-up of abnormal BP (Table 7) [14]. No other health centre or individual characteristics showed significant associations with follow-up of abnormal results.

## Discussion

There is substantial room to improve the quality of preventive care to Indigenous adults in many locations across Australia - in terms of overall delivery of services, in delivery of a range of specific services and in follow up of abnormal findings from routine health checks. Overall, it appears that about one third of the recommended preventive services were delivered to clients in participating health centres. Variation in overall delivery of guideline scheduled services between health centres is striking, with the lowest adherence to delivery being 5% and the highest being 74%. For specific important measures such as BP screening, overall 71% of adults have a record within the previous two years. However the variation between centres of 23-100% reveals a critical requirement for action in some health centres. The generally small proportion of clients with records of or plans for follow-up of abnormal clinical findings among these 'well' adults also highlights an important area for improvement.

Limitations of this study include: 1) Health centres were not randomly selected and their participation in the project was on a voluntary basis and enrolment was staggered over a period of some years. Therefore, these data are not representative for the regions involved and differences between health centres may be partly the result of introduction of new policies over time. A longitudinal analysis including services with more than three years of data through participating in this project will be reported separately. 2) We relied on clinical medical records to retrieve preventive care data, which may underestimate actual service delivery if delivered services are not recorded in clinical records. While failure to document services may mean that services are delivered at higher levels than reflected in our data, the failure to document delivered services is itself a significant barrier to continuity and coordination of care and in preventing duplication and over servicing - especially in areas of high workforce turnover. Failure to document delivered services is therefore in itself a deficiency in quality of care. 3) The unweighted age and sex stratified random samples are designed to facilitate analysis of quality of care between communities. Estimates based on this sampling approach may differ from sampling approaches designed to provide population estimates.

The pattern of delivery of different services (with blood pressure checks and blood glucose testing for well adults being relatively high, followed by delivery of urinalysis, pap smear and STI screening, provision of brief interventions/counselling related to lifestyle change, and with lowest levels of delivery for oral health checks and counselling on emotional well being) to some extent reflects a gradient in strength of evidence for the preventive services specified in best practice guidelines [2,15]. Practitioners appear less likely to provide some services where the availability of referral services (e.g. for dental and mental health care [16]) is limited. However, the low proportion of adults identified as smokers in relation to known smoking rates in these communities is an example of an important gap in documentation of major risk factors where there are relatively simple primary care interventions with a reasonably well established evidence base.

The comparability of these findings with similar studies [17,18] is limited by the inclusion in these studies of the general adult population, while our study focuses on an age and sex stratified (unweighted) random samples of well adults. People with chronic illness are likely to have increased contact with the health systems and more opportunities for receiving preventive services, so delivery of preventive services to well adults might be expected to be lower than for people with chronic illness. As indicated above, the stratified random sample used in our study may also not be representative of the study populations in each community or of the study populations for all communities combined. Bearing these restrictions on comparability with other studies in mind, we note that delivery of some services (such as BP) in our study population compares reasonably well. However, the generally low levels of delivery of care, the well known burden of chronic disease in this population, the importance of early detection and treatment, and the high rates of attendance by our study population at primary care centres mean that many important opportunities are being missed and there is clearly a need for better delivery of preventive services.

High prevalence of health problems among "healthy adults" and low follow-up of identified problems are of significant concern. Similar to previous reports from Indigenous primary care settings [7,8], about 20%-40% of the participating "healthy adults" in our study had abnormal blood pressure, abnormal blood sugar levels, or proteinuria. This highlights the importance and necessity of systematically implementing preventive care for adults in Indigenous communities for early detection and management of preventable chronic disease. A parallel priority in preventive care is to effectively follow up and manage the abnormal conditions identified. Failure to follow up and implement management plans means resources and efforts invested in regular checking and screening of well adults are wasted and cannot be translated into improved health outcomes.

Our analysis of variation in preventive care indicates health centre level and individual client level factors have a similar level of influence on delivery of preventive care. The finding that accreditation of services is not clearly associated with quality of care is consistent with other research on this topic [19,20], and indicates the need for a more active approach to quality improvement (e.g. routine use of clinical data to monitor and improve quality of care; ongoing engagement of health centre staff in service planning, system redesign and implementation of improvement initiatives). The finding that delivery of preventive services is better in remote locations and worse in health centres with large service populations is likely to be at least partly due to greater use of a number of different providers by clients living in non-remote settings or larger centres. The finding that delivery of preventive services is better in community controlled services than government managed services supports the contention that community control (through its philosophy, organisation or funding) facilitates quality and access to care [21].

The substantial proportion of variation in preventive care attributable to client level factors points to the importance of health centre systems to deliver care in a way that most effectively meets the varying needs of individual clients. Regarding client level factors, we only collected demographic information of participants (i.e. age, sex and Indigenous status). Male participants appeared less likely to access preventive services than females in our study. This may reflect the perceptions of many Indigenous men who consider health centres as "women's places" [22], as health centres in remote communities are predominantly staffed by females. Gender appropriate workforce and infrastructure may encourage Indigenous men to better use of health services. Other client level factors, such as their health literacy [23], perceptions of physical, social and cultural accessibility of the centre, and factors which influence a client's relationship with health centre staff [24], are important influences on the delivery or uptake of preventive care. These questions need to be investigated in future research, as well as associations of specific health centre system factors [6,25] with preventive care.

Beyond addressing potential health centre and client level factors, a supportive health policy has been recognised as critical to the implementation of preventive care to populations [26]. The introduction of a new Medicare item (item 710) in 2004 for health assessment of Indigenous people aged 15-54 years has been welcomed as an example of innovative policy in Indigenous health [27]. However, the impact of this measure is unclear and the longitudinal analysis of the ABCDE data should provide some evidence in this area. Previous research indicates that the Medicare rebates for providing preventive care may have less effect in motivating practitioners working in remote Indigenous community health centres who are usually in salaried positions [6]. More recent policy developments include new legislation which authorises practice nurses and Aboriginal Health Workers to access some Medicare items (e.g. for provision of immunisation and follow up services for Indigenous people after health assessment) [28], and introduction of the Indigenous Practice Incentives Program (PIP) to encourage population-based care [29]. Further refinement of health policies includes strengthening direct financial and workforce support to health centres based on needs of defined populations [30].

## Conclusions

There is great potential to improve delivery of preventive services to well adults in Indigenous primary care settings. Particular attention should be given to improving follow-up of abnormal clinical findings identified by preventive health assessments. The national collaborative approach that underpins the data presented in this paper provides a significant opportunity to advance understanding of variation in care and to develop and examine the effect of innovative strategies to enhance the quality of care for Indigenous Australians.

## Competing interests

The authors declare that they have no competing interests.

## Authors' contributions

RB played a lead role in conceptualisation of study design, development of measurement tools, project management, and revising of the manuscript. DS played a major role in reviewing the literature and conceptualisation, conducted data analysis, and drafted the manuscript. CC, AB, TW and ST and HB contributed to study design and facilitated engagement of health services. MD and LO contributed to study design and development of measurement tools. MD, LO, RK, CK, RC, HL, JH carried out field work and conducted data collection. All authors contributed to the interpretation of findings, read and approved the final manuscript.

## Pre-publication history

The pre-publication history for this paper can be accessed here:

http://www.biomedcentral.com/1472-6963/11/139/prepub

## References

[B1] Australian Institute of Health and Welfare Chronic diseases and associated risk factors in Australia 2006 Canberra: AIHW

[B2] National Aboriginal Community Controlled Health Organisation National guide to a preventive health assessment in Aboriginal and Torres Strait Islander peoples 2005 Melbourne: Royal Australian College of General Practitioners

[B3] Australian Government Department of Health and Ageing: MBS Primary Care Items Medicare Health Assessment for Aboriginal and Torres Strait Islander People - Fact Sheet http://www.health.gov.au/internet/main/publishing.nsf/Content/mbsprimarycare_ATSI_MBSitem715 Accessed on 13/09/2010

[B4] Australian Government Preventative Health Taskforce Australia: The Healthiest Country by 2020 - National Preventative Health Strategy - Overview 2009 Canberra: Australian Government Preventative Health Taskforce

[B5] Bailie RS Togni SJ Si D Robinson G d'Abbs PH Preventive medical care in remote Aboriginal communities in the Northern Territory: a follow-up study of the impact of clinical guidelines, computerised recall and reminder systems, and audit and feedback BMC Health ServRes 2003 3 1 15 10.1186/1472-6963-3-15 PMC194217 12890291

[B6] Si D Bailie RS Dowden M O'Donoghue L Connors C Robinson GW Cunningham J Condon JR Weeramanthri TS Delivery of preventive health services to Indigenous adults: response to a systems-oriented primary care quality improvement intervention Med J Aust 2007 187 8 453 457 17937642 10.5694/j.1326-5377.2007.tb01356.x

[B7] Miller G McDermott R McCulloch B Leonard D Arabena K Muller R The Well Person's Health Check: a population screening program in indigenous communities in north Queensland Aust Health Rev 2002 25 6 136 147 10.1071/AH020136b 12536873

[B8] Spurling GK Hayman NE Cooney AL Adult health checks for Indigenous Australians: the first year's experience from the Inala Indigenous Health Service Med J Aust 2009 190 10 562 564 19450202 10.5694/j.1326-5377.2009.tb02563.x

[B9] Kelaher M Dunt D Thomas D Anderson I Comparison of the uptake of health assessment items for Aboriginal and Torres Strait Islander people and other Australians: implications for policy Aust New Zealand Health Policy 2005 2 21 10.1186/1743-8462-2-21 16150154 PMC1239906

[B10] Bailie R Si D Connors C Weeramanthri T Clark L Dowden M O'Donohue L Condon J Thompson S Clelland N Nagel T Gardner K Brown A Study protocol: Audit and Best Practice for Chronic Disease Extension (ABCDE) Project BMC Health Serv Res 2008 8 184 10.1186/1472-6963-8-184 18799011 PMC2556328

[B11] Central Australian Rural Practitioner Association CARPA standard treatment manual: a clinic manual for primary health care practitioners in remote and rural communities in Central and Northern Australia 2003 Alice Springs: Central Australian Rural Practitioner Association

[B12] StataCorp Stata longitudinal/panel data reference mmanual. Release 10 2007 College Station, TX: StataCorp LP

[B13] Snijders TAB R J Multilevel analysis: an introduction to basic and advanced multilevel modeling 1999 Thousand Oaks, CA: Sage

[B14] Zhang J Yu KF What's the Relative Risk?: A Method of Correcting the Odds Ratio in Cohort Studies of Common Outcomes JAMA: The Journal of the American Medical Association 1998 280 19 1690 1691 10.1001/jama.280.19.1690 9832001

[B15] The National Preventive and Community Medicine Committee of the Royal Australian College of General Practitioners Guidelines for preventive activities in general practice Aust Fam Physician 2002 31 S1 S61

[B16] Australian Bureau of Statistics, Australian Institute of Health and Welfare The health and welfare of Australia's Aboriginal and Torres Strait Islander peoples, 2008 2008 Canberra: Commonwealth of Australia21624192

[B17] McGlynn EA Asch SM Adams J Keesey J Hicks J DeCristofaro A Kerr EA The quality of health care delivered to adults in the United States NEnglJMed 2003 348 26 2635 2645 10.1056/NEJMsa022615 12826639

[B18] Schoen C Osborn R Huynh PT Doty M Davis K Zapert K Peugh J Primary care and health system performance: adults' experiences in five countries Health Aff (Millwood) 2004 Suppl Web Exclusives W4-487 503 10.1377/hlthaff.w4.487 15513956

[B19] Greenfield D Braithwaite J Health sector accreditation research: a systematic review Int J Qual Health Care 2008 20 3 172 183 10.1093/intqhc/mzn005 18339666

[B20] Ovretveit J Gustafson D Using research to inform quality programmes BMJ 2003 326 7392 759 761 10.1136/bmj.326.7392.759 12676849 PMC1125660

[B21] National Aboriginal and Torres Strait Islander Health Council (NATSIHC) National Strategic Framework for Aboriginal and Torres Strait Islander Health: Framework for action by Governments 2003 Canberra: NATSIHC

[B22] McCoy B Holding Men: Kanyirninpa and the health of Aboriginal men 2008 Canberra: Aboriginal Studies Press

[B23] Smylie J Williams L Cooper N Culture-based literacy and Aboriginal health Can J Public Health 2006 97 Suppl 2 S21 25 16805157 10.1007/BF03405369 PMC6975592

[B24] Foster CH What nurses should know when working in Aboriginal communities Can Nurse 2006 102 28 31 16734350

[B25] Glasgow RE Orleans CT Wagner EH Does the chronic care model serve also as a template for improving prevention? Milbank Q 2001 79 4 579 612 iv-v 10.1111/1468-0009.00222 11789118 PMC2751207

[B26] Couzos S Murray R Aboriginal primary health care: an evidence-based approach 2008 Melbourne: Oxford University Press

[B27] Mayers NR Couzos S Towards health equity through an adult health check for Aboriginal and Torres Strait Islander people: an important Australian initiative that sets an international precedent Med J Aust 2004 181 10 531 532 15540961

[B28] Medicare Australia Nurse practitioners and midwives http://www.medicareaustralia.gov.au/provider/other-healthcare/nurse-midwives.jsp accessed 14 September 2010.

[B29] Medicare Australia Practice Incentives Program (PIP) - Indigenous Health Incentive http://www.medicareaustralia.gov.au/provider/incentives/pip/files/2864-pip-indigenous-health-incentive-guidelines-5-5-11.pdf Accessed 30 May 2011.

[B30] Australian Government Preventative Health Taskforce Australia: The Healthiest Country by 2020 - National Preventative Health Strategy - the roadmap for action 2009 Canberra: Australian Government Preventative Health Taskforce

